# Expanding the Antimalarial Drug Arsenal—Now, But How?

**DOI:** 10.3390/ph4050681

**Published:** 2011-04-26

**Authors:** Brian T. Grimberg, Rajeev K. Mehlotra

**Affiliations:** Center for Global Health and Diseases, School of Medicine, Case Western Reserve University, Cleveland, OH 44106, USA; E-Mails: brian.grimberg@case.edu (B.T.G.); rajeev.mehlotra@case.edu (R.K.M.); Tel.: +1-216-368-6328 or +1-216-368-6172, Fax: +1-216-368-4825

**Keywords:** malaria, falciparum, artemisinin resistance, natural products, drug discovery, kinases, HDAC, DHODH

## Abstract

The number of available and effective antimalarial drugs is quickly dwindling. This is mainly because a number of drug resistance-associated mutations in malaria parasite genes, such as *crt*, *mdr1*, *dhfr*/*dhps*, and others, have led to widespread resistance to all known classes of antimalarial compounds. Unfortunately, malaria parasites have started to exhibit some level of resistance in Southeast Asia even to the most recently introduced class of drugs, artemisinins. While there is much need, the antimalarial drug development pipeline remains woefully thin, with little chemical diversity, and there is currently no alternative to the precious artemisinins. It is difficult to predict where the next generation of antimalarial drugs will come from; however, there are six major approaches: (i) re-optimizing the use of existing antimalarials by either replacement/rotation or combination approach; (ii) repurposing drugs that are currently used to treat other infections or diseases; (iii) chemically modifying existing antimalarial compounds; (iv) exploring natural sources; (v) large-scale screening of diverse chemical libraries; and (vi) through parasite genome-based (“targeted”) discoveries. When any newly discovered effective antimalarial treatment is used by the populus, we must maintain constant vigilance for both parasite-specific and human-related factors that are likely to hamper its success. This article is neither comprehensive nor conclusive. Our purpose is to provide an overview of antimalarial drug resistance, associated parasite genetic factors (1. Introduction; 2. Emergence of artemisinin resistance in *P. falciparum*), and the antimalarial drug development pipeline (3. Overview of the global pipeline of antimalarial drugs), and highlight some examples of the aforementioned approaches to future antimalarial treatment. These approaches can be categorized into “short term” (4. Feasible options for now) and “long term” (5. Next generation of antimalarial treatment— Approaches and candidates). However, these two categories are interrelated, and the approaches in both should be implemented in parallel with focus on developing a successful, long-lasting antimalarial chemotherapy.

## 1. Introduction

### 1.1. Malaria

More than 40% of the world's population, much of it socioeconomically and politically challenged, live in areas where malaria, alone or together with HIV/AIDS and tuberculosis, is a significant health risk [1,2]. According to the World Health Organization (WHO), approximately 250 million clinical cases of malaria occur every year [3]. Malaria is estimated to kill nearly one million people annually, with most of the deaths occurring in children under 5 years of age in sub-Saharan Africa [4]. If children survive multiple infections, such exposure leads to a natural immunity that limits the severity of the disease later in life. However, this immunity wanes in the absence of continued exposure to malaria infections. Additionally, pregnant women and newborns have reduced immunity, and therefore are vulnerable to severe complications of malaria infection and disease [5,6].

Malaria is primarily caused by four species of the protozoan parasite *Plasmodium: P. falciparum*, *P. vivax*, *P. malariae*, and *P. ovale*, which are transmitted by over 70 species of *Anopheles* mosquitoes [7,8]. These parasite species occur sympatrically both in human populations and within infected individuals, with *P. falciparum* and *P. vivax* being the predominant species [9-11]. Approximately 80% of all malaria cases and 90% of malaria-attributed deaths occur in Africa, and are caused by *P. falciparum* [3]. Outside of Africa, *P. vivax* is the most widespread species, occurring largely in Asia, including the Middle East and the Western Pacific, and in Central and South America [12]. This parasite species causes a relatively less lethal form of the disease compared with *P. falciparum* [12,13].

### 1.2. Overview of Antimalarial Drugs

Today when many mosquito vectors are resistant to insecticides [14,15], and an effective vaccine is not yet available [14,16], chemoprophylaxis/chemotherapy remains the principal means of combating malaria. Over the past 60 to 70 years, since the introduction of synthetic antimalarials, only a small number of compounds, belonging to three broad classes, have been found suitable for clinical usage [17,18]. These classes are described below.

#### 1.2.1. Quinine and related drugs

Quinine, originally extracted from cinchona bark in the early 1800s, along with its dextroisomer quinidine, is still one of the most important drugs for the treatment of uncomplicated malaria, and often the drug of last resort for the treatment of severe malaria. Chloroquine (CQ), a 4-aminoquinoline derivative of quinine, has been the most successful, inexpensive, and therefore the most widely used antimalarial drug since the 1940s. However, its usefulness has rapidly declined in those parts of the world where CQ-resistant strains of *P. falciparum* and *P. vivax* have emerged and are now widespread. Amodiaquine, an analogue of CQ, is a pro-drug that relies on its active metabolite monodesethylamodiaquine, and is still effective in areas of Africa, but not in regions of South America. Other quinine-related, commonly used drugs include mefloquine, a 4-quinoline-methanol derivative of quinine, and the 8-aminoquinoline derivative, primaquine; the latter is specifically used for eliminating relapse causing, latent hepatic forms (hypnozoites) of *P. vivax*. Halofantrine and lumefantrine, both structurally related to quinine, were found to be effective against multidrug-resistant falciparum malaria [17,19,20]. While lumefantrine, in combination with an artemisinin derivative, artemether (Coartem), is recommended by the WHO for treating uncomplicated falciparum malaria, halofantrine generally is not recommended because of serious side effects and extensive cross-resistance with mefloquine [21].

#### 1.2.2. Antifolate combination drugs

Antifolate drugs include various combinations of dihydrofolate reductase enzyme (DHFR) inhibitors, such as pyrimethamine, proguanil, and chlorproguanil, and dihydropteroate synthase enzyme (DHPS) inhibitors, such as sulfadoxine and dapsone. With rapidly growing sulfadoxine-pyrimethamine (SP) resistance, a new combination drug, Lapdap (chlorproguanil-dapsone), was tested in Africa in the early 2000s, but was withdrawn in 2008 because of hemolytic anemia in patients with glucose-6-phosphate dehydrogenase enzyme (G6PD) deficiency [22].

#### 1.2.3. Artemisinin and its derivatives

Artemisinin drugs, which originated from the Chinese herb qing hao (*Artemisia annua*), belong to a unique class of compounds, the sesquiterpene lactone endoperoxides [18]. The parent compound of this class is artemisinin (quinghaosu), whereas dihydroartemisinin (DHA), artesunate, artemether, and β-arteether are the most common derivatives of artemisinin; DHA is the main bioactive metabolite of all artemisinin derivatives (artesunate, artemether, β-arteether, *etc.*), and is also available as a drug itself [17,18,20].

Since 2001, the WHO has recommended the use of artemisinin-based combination therapies (ACTs) for treating falciparum malaria in all countries where resistance to monotherapies or non-artemisinin combination therapies (e.g., SP) is prevalent [23]. The rationale for the use of ACTs is based on the facts that artemisinin derivatives are highly potent and fast acting, and that the partner drug in ACT has a long half-life, which allows killing the parasites that may have escaped the artemisinin inhibition. Thus, it is thought that ACTs will delay the onset of resistance by acting as a “double-edged sword” [18,24,25]. ACTs, such as artemether-lumefantrine, artesunate-amodiaquine, and artesunate-mefloquine are being used in China, Southeast Asia, many parts of Africa, and some parts of South America [17,18,20]. Introduction of ACTs has initiated noticeable reduction in malaria prevalence in these endemic regions of the world [18]. Although the mechanism(s) of action is poorly understood (described below), the current high level of interest in artemisinin drugs is due to their well-recognized pharmacological advantages: These drugs act rapidly upon asexual blood stages of CQ-sensitive as well as CQ-resistant strains of both *P. falciparum* and *P. vivax*, and reduce the parasite biomass very quickly, by about 4-logs for each asexual cycle. In addition, these drugs are gametocytocidal. Thus, through rapid killing of asexual blood stages and developing gametocytes, artemisinin drugs significantly limit the transmission potential of the treated infections. These drugs have large therapeutic windows, and based on extensive human use, they appear to be safe, even in children and mid/late pregnant women. Furthermore, there is no reported “added toxicity” when these drugs are used in combination with other types of antimalarial compounds.

In addition to these three main classes of compounds, the antibiotic tetracycline and its derivatives, such as doxycycline, are consistently active against all species of malaria, and in combination with quinine, are particularly useful for the treatment of severe falciparum malaria [17]. Until recently, a combination of atovaquone, a hydroxynaphthoquinone, and proguanil (Malarone) was considered to be effective against CQ- and multidrug-resistant falciparum malaria; atovaquone resistance has recently been noticed in Africa [26]. Piperaquine, another member of the 4-aminoquinoline group, in combination with DHA (Artekin), holds the promise of being successful in CQ-resistant endemic areas of Southeast Asia [17,18,20].

### 1.3. Overview of Antimalarial Drug Resistance

This limited antimalarial armament is now severely compromised because of the parasite's remarkable ability to develop resistance to these compounds [19,27]. In many different malaria-endemic areas, low to high-level resistance in the predominant malaria parasites, *P. falciparum* and *P. vivax*, has been observed for CQ, amodiaquine, mefloquine, primaquine, and SP. *Plasmodium falciparum* has developed resistance to nearly all antimalarial drugs in current use, although the geographic distribution of resistance to any one particular drug varies greatly. In particular, Southeast Asia has a highly variable distribution of falciparum drug resistance; some areas have a high prevalence of complete resistance to multiple drugs, while elsewhere there is a spectrum of sensitivity to various drugs [19]. Until 2009, no noticeable clinical resistance to artemisinin drugs was reported. However, as described below, a number of recent studies have raised concerns about the efficacy of ACTs, particularly in Southeast Asia.

### 1.4. Overview of Genetic Basis for Antimalarial Drug Resistance

It is believed that the selection of parasites harboring polymorphisms, particularly point mutations, associated with reduced drug sensitivity, is the primary basis for drug resistance in malaria parasites [28,29]. Drug-resistant parasites are more likely to be selected if parasite populations are exposed to sub-therapeutic drug concentrations through (a) unregulated drug use; (b) the use of inadequate drug regimens; and/or (c) the use of long half-life drugs singly or in non-artemisinin combination therapies. In recent years, significant progress has been made to understand the genetic/molecular mechanisms underlying drug resistance in malaria parasites [30,31]. Chloroquine resistance (CQR) in *P. falciparum* is now linked to point mutations in the chloroquine resistance transporter (PfCRT [encoded by *pfcrt*, located on chromosome 7]). *Pfcrt*-K76T mutation confers resistance *in vitro*, and is the most reliable molecular marker for CQR. Polymorphisms, including copy number variation and point mutations, in another parasite transporter, multidrug resistance (PfMDR1 or Pgh1 [encoded by *pfmdr1*, located on chromosome 5]), contribute to the parasite's susceptibility to a variety of antimalarial drugs. Point mutations in *pfmdr1* play a modulatory role in CQR, which appears to be a parasite strain-dependent phenomenon [32].

Point mutations in the *P. falciparum* DHPS enzyme (encoded by *pf-dhps*, located on chromosome 8) are involved in the mechanism of resistance to the sulfa class of antimalarials, and accumulation of mutations in the *P. falciparum* DHFR domain (encoded by *pf-dhfr*, located on chromosome 4) defines the major mechanism of high-level pyrimethamine resistance. In field studies, a *pf-dhps* double mutant (437G with either 540E or 581G), combined with the *pf-dhfr* triple mutant (108N_51I_59R), was found to be frequently associated with SP treatment failure [28,29]. Orthologues of *pfcrt* (*pvcrt-o*), *pfmdr1* (*pvmdr1*), *pf-dhps* (*pv-dhps*), and *pf-dhfr* (*pv-dhfr*) in *P. vivax* have been identified, and found to be polymorphic. However, associations of the mutant alleles of *pvcrt-o*/*pvmdr1* and *pv-dhps*/*pv-dhfr* with clinical resistance to CQ and SP, respectively, are unclear [33].

## 2. Emergence of Artemisinin Resistance in *P. falciparum*

A major advance in the search for effective treatment for drug-resistant malaria came with the discovery of artemisinin and its derivatives. These compounds show very rapid parasite clearance times and faster fever resolution than any other currently licensed antimalarial drug [18]. Given the recent significant impact of ACTs on malaria morbidity and mortality, it is worrisome that higher recrudescence rates of *P. falciparum* malaria after artemisinin treatment are seen in some areas. Recrudescence, the reappearance of an infection after a period of quiescence, occurs in up to 30% of patients on artemisinin monotherapy, and in up to 10% of patients on ACTs [18,34]. The underlying mechanism of recrudescence after artemisinin treatment is unclear. As illustrated by recent *in vitro* studies, the occurrence of parasite dormancy, where parasites enter a temporary growth-arrested state, may provide a plausible explanation for this phenomenon [35,36].

Furthermore, there are recent concerns that the efficacy of ACTs has declined near the Thai-Cambodia border, a historical “hot spot” for emergence and evolution of multidrug-resistant malaria parasites [37]. In this region, artemisinin resistance is characterized by significantly slower parasite clearance *in vivo*, with or without corresponding reductions on conventional *in vitro* susceptibility testing [38,39]. A recent heritability study found that the observed artemisinin-resistant phenotype of the parasites has a genetic basis [40]. The study showed that genetically identical parasite strains, defined by microsatellite typing, strongly clustered in patients with slow *versus* faster parasite clearance rates. It was suggested that the artemisinin-resistant phenotype is expected to spread within parasite populations that live where artemisinins are deployed unless associated fitness costs of the putative resistance mutation(s) outweigh selective benefits. The genetic basis for artemisinin resistance is not known at present. The main reason for this lack of knowledge is that the molecular mechanism(s) of action of artemisinin drugs remains uncertain and debatable, although a number of potential targets have been proposed [41,42]. Investigations into potential protein targets of artemisinin drugs have included PfATP6, a *P. falciparum* SERCA-type calcium-dependent ATPase in the endoplasmic reticulum [43]; PfCRT; PfMDR1; *P. falciparum* multidrug resistance-associated protein 1 (PfMRP1), residing on the parasite plasma membrane; a putative deubiquitinating protease termed UBP-1; and mitochondrial proteins [41,42]. A recent study, conducted on artemisinin-resistant *P. falciparum* strains from western Cambodia, did not find any correlation between artemisinin-resistant phenotype and *pfmdr1* mutations/amplification, as well as mutations in *pfatp6* (or *pfserca*), *pfcrt*, *ubp-1*, or mtDNA [44].

At present there is no concrete evidence of artemisinin resistance occurring elsewhere. Preliminary reports of emerging artemisinin resistance in South America and some African countries are as yet unconfirmed. For now, the situation appears to be confined to the Thai-Cambodia border, and is most likely a result of distinctive features of malaria treatment in this geographic area. These include unregulated artemisinin monotherapy for more than 30 years, creating massive drug pressure, and counterfeited or substandard tablets that contain less active ingredients than stated [45]. However, if the problem is not urgently and appropriately addressed, it is feared that artemisinin resistance could spread worldwide. Given the pivotal role that artemisinin drugs now play, this would be a major blow for malaria treatment and control, since there are currently no alternative drugs to replace them. Even the development pipeline of new drugs consists predominantly of ACTs.

## 3. Overview of the Global Pipeline of Antimalarial Drugs

Olliaro and Wells [46] reviewed the global pipeline of new antimalarial combinations and chemical entities, in various stages of development till February 2009. Overall, the pipeline consists of 22 projects, either completed (artemether-lumefantrine and artesunate-amodiaquine), in various clinical stages (phase III/registration stage, n = 4; phase II, n = 8; phase I, n = 5), or in preclinical translational stage (n = 3). Chemical novelty is relatively low among the products in clinical stages of development; the prevailing families are of artemisinin-type and quinoline compounds, mostly in combination with each other. Among the non-artemisinin and/or non-quinoline combinations and single compounds are: Azithromycin-CQ (phase III), fosmidomycin-clindamycin (phase II), methylene blue-amodiaquine (phase II), SAR 97276 (a choline uptake antagonist, phase II), and tinidazole (an anti-parasitic nitroimidazole compound, phase II).

It is clear from the size of the antimalarial drug development pipeline that the pipeline has not reached critical mass, which is of concern particularly when we consider the recent emergence of artemisinin resistance, and the apparent decrease in time to resistance to each new drug/drug combination. The challenges for now and for the future are: (i) how to ensure that we have compounds to combat emerging and future antimalarial drug resistance; and (ii) how to initiate and expedite the development of compounds that are as innovative as possible with respect to their chemical scaffold and molecular target.

## 4. Feasible Options for Now

### 4.1. Re-Optimizing Existing Antimalarials

#### 4.1.1. Replacing/rotating drugs—Examples of CQ and SP

Recently, a drug replacement/rotation approach has been suggested to stem the tide of resistance by changing the “playing field” for malaria parasites [47,48]. This approach is based on the understanding that the drug-resistant mutant forms are likely to be less fit than their wild-type strains in the absence of drug selection [49]. It has been shown that when CQ was withdrawn from Malawi, where a high level of CQR prevailed, CQ sensitivity returned [50]. The return of CQ-sensitive falciparum malaria in Malawi took almost 10 years, and was associated with a re-expansion of *pfcrt*-76K allele-carrying diverse, sensitive parasites [51,52]. Similar association between cessation of CQ use and decrease in *pfcrt*-76T allele-carrying resistant parasites has been observed in Kenya [53] as well as in China [54,55]. However, it is estimated that in these areas, sensitivity to CQ may take many more years to return to a level where the drug could be used effectively again [53,54].

Regarding SP, it has been shown that in the Amazon region of Peru, the frequencies of the mutant *pf-dhfr* and *pf-dhps* alleles, conferring SP resistance, declined significantly 5 years after the drug was replaced [48]. This, however, was not the case in Venezuela [56] and Cambodia [57], where the SP resistance-conferring alleles have remained at a high frequency 8 years and 2 decades, respectively, after the replacement of SP. It appears that the outcome of the drug replacement/rotation approach is not universal but malaria-endemic region dependent; a number of diverse ecological and epidemiological factors are likely to play a role in determining the outcome of this approach. In addition, the logistics, including the cost component, of this approach may pose challenges for some countries.

#### 4.1.2. Combining drugs—Examples of CQ-CQR reversers and multiple-drug therapy

Improving the use of existing antimalarial drugs by designing new combinations is another potential approach to combat resistance. CQ entered widespread usage in the 1940s [19]. In spite of widespread resistance, this drug is still widely available because it is inexpensive and relatively non-toxic. In a thought-provoking article, Ginsburg [58] suggested that differential use of CQ in holoendemic areas that precludes children and pregnant women could still confer an efficacious protection for semi-immune adults, and more efficient treatment protocols could be devised to treat even patients infected with CQ-resistant parasite strains. According to the author [58], since the antimalarial activity of CQ is pleiotropic, drug resistance may be due to different mechanisms, each amenable to reversal by drug combination. Since the pivotal study by Martin and colleagues [59], which showed that CQR in *P. falciparum* was partly reversible by verapamil, it has been envisioned that the usefulness of CQ can be rescued by attempting to reverse the resistance—By co-administering CQ with another drug that chemosensitizes the parasite to its effect [60-64]. Among more than 40 such compounds, only one, chlorpheniramine, a histamine H1 receptor antagonist, in combination with CQ has been tested in patients with some success [58,60]. Of further interest, primaquine and antiretroviral protease inhibitors (PIs) saquinavir, ritonavir, and indinavir, may act as CQR reversers [60]. Fluoxetine, an antidepressant of the selective serotonin reuptake inhibitor class, has been shown to reverse both CQ and mefloquine resistance, while ketoconazole, a synthetic antifungal drug of the imidazole class, has been shown to reverse mefloquine resistance in a monkey model [60]. In addition, novel compounds that are more potent and less toxic than the archetypal CQR reverser verapamil are under investigations [60]. Thus, the reports of new resistance reversers over the last few years have generated considerable interest, and may lead to an effective stopgap therapy for drug-resistant malaria.

Use of combination therapy is currently mandated by the WHO [23]. With widespread high-level CQR, SP, as a 2-drug combination, was a successful first-line treatment in Africa for almost two decades. Currently, our hope rests mainly upon ACTs and atovaquone-proguanil (Malarone). As mentioned before, it is of concern that some of these combinations have started showing signs of failure. A further approach to minimize/delay the development of drug resistance in malaria is to apply combinations of three or more drugs (“multiple-drug therapy”), as is practiced for treating HIV/AIDS and tuberculosis. Attempts at combining existing drugs with CQ (e.g., CQ-SP) have proved less successful, whereas amodiaquine-SP has shown encouraging clinical activity [65]. Recently, a triple combination of artesunate-Lapdap, despite the side effects associated with Lapdap, is under evaluation for the treatment of uncomplicated falciparum malaria in Africa [66]. A limitation of this approach is the increased possibility of drug-drug interactions or the need for a reformulation of the drugs, both of which can prove costly and time-consuming to resolve. Furthermore, given that only a handful of effective antimalarial drugs are now left, this approach seems less promising.

#### 4.1.3. (Re) Considering drugs with limitations—Example of tafenoquine

With any drug, one of the major limitations is side effects. Mefloquine was first synthesized in 1969 primarily for the purpose of chemoprophylaxis in the U.S. military following the then recently discovered threat of CQ-resistant falciparum malaria. The drug has been in use as monotherapy and as combination therapy with either SP or artesunate. However, due to increasing incidences of resistance, particularly in the areas of multidrug resistance [67,68], newer information about toxicity [69,70], and the potential for severe neuropsychiatric adverse events among US military personnel [71], doxycycline is considered as a replacement for mefloquine chemoprophylaxis [72]. Although doxycycline has scanty side effects, a major limitation is that in prophylactic therapy, it must be taken daily leading to high non-compliance. Therefore, a new investigational drug, tafenoquine, an analogue of primaquine, related to mefloquine, was introduced in 1990s [73,74]. Although like primaquine, tafenoquine causes hemolysis in patients with G6PD deficiency, the drug has the potential advantage of a shorter treatment course, and therefore increased patient compliance, and higher therapeutic index than primaquine [73-75]. Thus, the interest in tafenoquine has been continued and efforts are directed at ameliorating side effects, so that this new efficacious compound can be used for malaria prevention as well as treatment [76-78].

### 4.2. Repurposing Drugs Used for Other Infections or Diseases

One of the approaches for rapidly identifying and bringing new drugs to the market for the treatment of malaria is to repurpose existing drugs that are currently approved for treatment of other infections or diseases.

#### 4.2.1. Drugs used for other infections—Anti-HIV/AIDS drugs

It is known that some antimalarial drugs have moderate anti-HIV activities [79,80]. However, recent studies have also demonstrated that certain antiretroviral agents can inhibit malaria parasite growth. Skinner-Adams *et al.* [81] showed that antiretroviral PIs saquinavir, ritonavir, and indinavir were effective against *P. falciparum*. Subsequently, other antiretroviral PIs were also shown to exhibit potent antimalarial activity [82,83]. The current working hypothesis for the antimalarial activity of antiretroviral PIs is that these compounds inhibit one or more of the parasite's aspartic proteases (termed plasmepsins), located on the food/digestive vacuole or non-digestive vacuole [84-86].

Recently, we found that a non-nucleoside reverse transcriptase inhibitor, TMC-125, which had been shown to be highly active against HIV [87], demonstrated potent activity against *P. falciparum* [88]. It is theorized that this compound targets the parasite enzyme PfTERT, a catalytic reverse transcriptase component of telomerase, expressed in asexual blood-stage parasites that have begun DNA synthesis [89]. There appears to be only one gene for TERT in *P. falciparum* [89], *P. vivax* [90], and *P. knowlesi* [89], containing some highly conserved regions across species [89,90]. Since telomerase activity is likely to be necessary during blood-stage parasite proliferation, it is hoped that screening other reverse transcriptase inhibitors, or designing specific anti-telomerase compounds may lead to novel antimalarial drugs with potent activity against multiple-species infections.

The potential for crossover between malaria and HIV treatments is undoubtedly intriguing, as most of the malaria-endemic areas also bear the brunt of the HIV pandemic. However, at a time when access to antiretroviral drugs is increasing, and new combinations of antimalarial drugs, particularly ACTs, are being evaluated, it is important that potential interactions between therapies for these 2 infections are also understood [79,80,91]. In this regard, it is important to mention that human drug-metabolizing enzymes CYP2B6 (phase I metabolism) and UGT2B7 (phase II metabolism) play important roles in the metabolism of antiretroviral drugs efavirenz, nevirapine, and zidovudine, as well as antimalarial artemisinin drugs, including their active metabolite DHA [92-94]. Functional polymorphisms in *CYP2B6* and *UGT2B7* are highly prevalent in the regions where HIV/AIDS and malaria co-occur [93-95]. Phenotypic consequences of polymorphisms in these enzyme genes on the pharmacokinetics and effectiveness of artemisinin drugs are yet to be determined, and comprehensive studies evaluating the potential interactions between these antiretroviral and antimalarial drugs in individuals with or without polymorphisms are needed.

#### 4.2.2. Drugs used for other diseases—Anti-cancer drugs

A new area in antimalarial drug discovery is the exploration of anti-cancer drugs that target cellular programs such as cell proliferation, cell differentiation, and cell death [88,96,97]. The interesting revelation that artesunate is effective against certain types of cancer, and may target one or more of these cellular programs [98-100], also lends credence to this path of investigation. Our recent study has shown that two anti-cancer compounds, SU-11274 and Bay 43-9006, exhibit potent activities (IC_50_ values <1 μM) against *P. falciparum* [88]. In humans, SU-11274 acts as a specific inhibitor of the MET receptor tyrosine kinase activity [101]. Bay 43-9006, a dual-action inhibitor, targets the RAF/MEK/ERK signaling pathway in tumor cells, and receptor tyrosine kinases VEGFR/PDGFR in tumor vasculature [102]. The specific targets for SU-11274 and Bay 43-9006 activities against *P. falciparum* are not clear yet, but with the analysis of *Plasmodium* spp. genomes, plasmodial protein kinases (“*Plasmodium* kinome”) are emerging as highly promising targets for such compounds [103,104]. In addition, inhibition of protein kinases of the human host is being viewed as another major parasiticidal strategy [97,103,105]. Recently, Doerig and colleagues [97] showed that the host PAK-MEK signaling pathway was selectively activated in *P. falciparum*-infected red blood cells. Selective inhibitors of PAK (IPA-3, IC_50_ value 2 μM) and MEK (U0126 [IC_50_ value 3 μM], PD184352 [IC_50_ value 7 μM]) inhibited the parasite proliferation. Moreover, the MEK inhibitors showed *in vitro* parasiticidal effects on hepatocyte and erythrocyte stages of the rodent malaria parasite *P. berghei*, indicating conservation of this subversive strategy in plasmodia [97].

As also discussed by Doerig and colleagues [97], several kinase-inhibiting drugs are now used clinically, mostly in a variety of cancers, with many more in different stages of clinical trials. Evaluating these inhibitors for antimalarial properties would considerably reduce the overall discovery/development cost and accelerate the process. A potential problem associated with most of these inhibitors is toxicity, as they tend to have small therapeutic indices. Most fatalities in malaria occur in children under 5, which emphasizes the need for not only highly efficacious but also very safe drugs. Although the kinase-inhibiting anti-cancer drugs have toxic effects, using them in malaria chemotherapy would require much shorter treatment periods, hopefully limiting the problem of toxicity. It would also be of interest to see whether these drugs can be used in combination with artemisinin drugs, which show no “added toxicity”, and whether such a combination helps in reducing the effective parasiticidal concentration of these drugs, thus reducing their toxicity.

## 5. Next Generation of Antimalarial Treatment—Approaches and Candidates

### 5.1. Development of New Drugs by Modifying Existing Compounds

#### 5.1.1. Derivatization

One method to synthesize new antimalarial drugs is to start with the chemical framework of existing antimalarial drugs; the 4- and 8-aminoquinolines and artemisinin drugs are the two common examples of this approach [106]. Recently, a variety of 4-aminoquinoline derivatives (thiourea, triazine, and bisquinoline) have been found to be highly active against *P. falciparum in vitro*, and against *P. yoelii* or *P. berghei* in mice [107-109]. Synthesis/screening of 4-position modified quinoline-methanol compounds is anticipated to yield an efficacious and less toxic replacement for mefloquine [110-112]. In addition to 4-aminoquinoline analogs, extensive derivatization approaches followed by better understanding of structure-activity relationships and biotransformation mechanisms of toxicity have also provided 8-aminoquinoline analogs with better pharmacologic and reduced toxicologic profiles [113].

Derivatives of artemisinin, particularly the water-soluble and efficacious artesunate, are another obvious choice for the synthesis of new compounds [20,114]. Although highly potent and fast acting, the currently available semisynthetic artemisinin derivatives are prone to hydrolysis, resulting in a short biological half-life and the generation of potentially toxic DHA [115,116]. Therefore, extensive efforts have been made to synthesize new derivatives of artemisinin and chemically-related compounds, which are more stable and exhibit potent *in vitro* [117,118] and *in vivo* [119-122] antimalarial activities. The *in vitro* activity of these new derivatives did not indicate any apparent cytotoxicity [118].

Thus, it appears that derivatization is one possible approach to prolong the clinical usefulness of aminoquinoline and artemisinin classes of compounds. However, as a potential limitation of this approach, toxicity and cross-resistance with the parent compound may still occur in some of the derivatives [123].

#### 5.1.2. Hybridization

Burgess *et al.* reported a highly innovative hybrid molecule that combines the pharmacophore of an active 4-amino-7-chloroquinoline antimalarial with a resistance-reversing group based on imipramine [124]. The compound was found to be highly active against both CQ-sensitive and CQ-resistant strains of *P. falciparum in vitro*, and against *P. chabaudi* in mice, with no obvious signs of toxicity. The authors named this class “reversed chloroquine (RCQ)”. Further studies showed that linking any of several reversal-agent-like moieties to a 4-amino-7-chloroquinoline yielded good parasiticidal activity [125]. Structure-activity relationship investigation indicated that RCQ molecules inhibit hemozoin formation in the parasite's digestive vacuole in a manner similar to that of CQ [126].

Recently, in a deliberate rational design of antimalarials acting specifically on multiple targets, several hybrid molecules have been developed in what has been termed “covalent bitherapy” [127]. One interesting report described the synthesis of a novel artemisinin-quinine hybrid with potent antimalarial activity [128]. Artemisinin was reduced to DHA and coupled to a carboxylic acid derivative of quinine via an ester linkage. This novel hybrid molecule had potent activity against CQ-sensitive and CQ-resistant strains of *P. falciparum in vitro*. The activity was superior to that of artemisinin alone, quinine alone, or a 1:1 mixture of artemisinin and quinine [128]. Another example is a trioxane motif of sesquiterpene lactone artemisinin covalently linked to a quinoline entity of an aminoquinoline (e.g., CQ), to form new modular molecules referred to as trioxaquines [127]. The trioxaquines had more improved antimalarial activity than their individual components, indicating a potential additive/synergistic effect of the hybrids, as well as good oral bioavailability and low toxicity [127]. Such hybrid molecules have the potential to delay or circumvent the development of resistance and reduce the risk of drug-drug adverse interactions, the latter often seen with multiple-drug therapies. The antimalarial activities, combined with a good drug profile (preliminary absorption, metabolism, and safety parameters) seem favorable for the selection of hybrid molecules for development as drug candidates [129].

### 5.2. Finding New Antimalarial Compounds by Exploring Natural Products

Historically, drug discovery and development has greatly benefited from sourcing compounds from nature. In fact, between 1981 and 2002, 61% of new chemical entities brought to the market can be traced back to, or were inspired by, natural sources [130]. Malaria drug discovery is no exception. The isolation of the antimalarial drug quinine from cinchona bark was accomplished in 1820. The bark had long been used by indigenous peoples in the Amazon region for the treatment of fevers. The Chinese herb qing hao (*Artemisia annua*) was also used as a treatment for fevers in China for more than 2,000 years, but it was not until 1972 that the active compound artemisinin was extracted, and later identified as a potent antimalarial drug. The 1990s saw a demise in research into natural products for drug discovery, due in part to the emergence of high-throughput screening and combinatorial chemistry. Today, however, the current demand for novel compounds to tackle emerging antimalarial resistance has stimulated new interest in their potential [131]. Several screening projects utilizing different natural sources, from the rainforest to the deep sea and from exotic microorganisms to plants, have been carried out, resulting in several interesting antimalarial lead compounds with remarkable chemical diversity [132-136]. Some such projects at the Medicines for Malaria Venture (MMV) are in the early-stage discovery pipeline [46].

The mode of action of compounds originating from natural products is mostly unknown, and, in order to understand the basis for their pharmacological effects, research focused on their synergistic or antagonistic interactions is needed [137]. It is also clear that the much desired success of this approach faces several challenges, such as species selection criteria, screening procedures, pharmacological models and fractionation processes, as well as prediction of clinical safety and efficacy [138,139]. Nevertheless, it is hoped that once the activity of natural medicinal products in humans is characterized, it can be used to identify new molecular scaffolds which will form the basis of the next generation of antimalarial therapies [140].

### 5.3. Finding New Antimalarial Drugs by Screening Diverse Chemical Libraries

#### 5.3.1. High-throughput screening

One of the major thrust areas for antimalarial drug discovery is to screen diverse chemical libraries using high-throughput assays. In recent years, impressive efforts by several drug-screening groups at pharmaceutical companies and academic institutions have generated a plethora of compounds, which may serve as new starting points for potential antimalarial drugs with novel mechanisms. Here, we summarize five such studies (Table 1).

(i) From a screen of ∼1.7 million compounds, Plouffe *et al.* [141] identified a diverse collection of ∼6,000 small molecules comprised of >530 distinct scaffolds, all of which showed potent antimalarial activity (<1.25 μM). Most known antimalarials were identified in this screen, thus validating their approach. In addition, the authors identified many novel chemical scaffolds, which likely act through both known and novel pathways.
(ii) Gamo *et al.* [142] screened nearly two million compounds in GlaxoSmithKline's chemical library for inhibitors of *P. falciparum*, of which 13,533 were confirmed to inhibit parasite growth by at least 80% at 2 μM concentration. More than 8,000 also showed potent activity against the multidrug resistant strain Dd2. Further analyses suggested several novel mechanisms of their antimalarial action, such as inhibition of protein kinases and host-pathogen interaction related targets. All of these proven plasmodial inhibitors, of which ∼11,000 (82%) were previously proprietary and thus unknown to the general research community, were made public to accelerate the pace of drug development for malaria.
(iii) Guiguemde *et al.* [143] used a phenotypic forward chemical genetic approach to assay 309,474 chemicals to discover new antimalarial chemotypes. The primary screen resulted in ∼1,300 hits with >80% activity against *P. falciparum* at 7 μM. Of these, antimalarial potencies of 172 compounds were re-confirmed to within tenfold by three laboratories using distinct methods providing the cross-validated hit set. A reverse chemical genetic study of this set identified 19 new inhibitors of four validated drug targets, and 15 novel binders among 61 malarial proteins. One exemplar compound (SJ000025081) at 100 mg kg^−1^ b.i.d. × 3 days resulted in a 90% suppression of *P. yoelii* parasitemia in mice.
(iv) From a library of ∼ 12,000 pure natural products and synthetic compounds with structural features found in natural products, Rottmann *et al.* [144] identified 275 primary hits with sub-micromolar activity against *P. falciparum*. After a series of analyses, a synthetic compound, related to the spiroazepineindole class, was selected for a medicinal chemistry lead optimization effort. Synthesis and evaluation of about 200 derivatives yielded the spirotetrahydro-β-carboline (or spiroindolone) compound NITD609. This compound displayed potent activity against a panel of *P. falciparum* strains (average IC_50_ range 0.5 to 1.4 nM), with no evidence of diminished potency against drug-resistant strains. The IC_50_ values against a clone of multidrug resistant strain Dd2, however, increased 7- to 24-fold (attaining a mean of 3 to 11 nM) after 3 to 4 months of constant drug pressure. Subsequent passaging of drug-selected parasites in drug-free media for 4 months showed no evidence of revertants, indicating that the resistance was stable. The resistance was associated with point mutations in the gene encoding the P-type cation-transporter ATPase4 *(pfatp4,* PFL0590c). Further safety and pharmacological preclinical evaluation of NITD609 is currently ongoing to support the initiation of human clinical trials.
(v) Using a high-throughput colorimetric assay for the detection of heme crystallization inhibitors, Rush *et al.* [145] identified 17 hit compounds out of 16,000 small molecules belonging to diverse structural classes. The IC_50_ values of these 17 hit compounds in the *P. falciparum* growth inhibition assay ranged from 0.2 μM to 19 μM, and the compounds belonged to structurally related pyrimidine and 1,3-benzoxathiol-2-one classes. These hit compounds are being tested against multidrug-resistant strains of *P. falciparum* to further confirm that they do not show cross-resistance with currently deployed heme crystallization inhibitors.

### 5.4. Virtual Screening

Virtual screening involves the rapid *in silico* assessment of large libraries of chemical structures in order to identify those which are most likely to bind to a drug target, typically a protein receptor or enzyme. This approach either alone or combined with high-throughput screening is gaining popularity in the antimalarial drug discovery area. Plouffe *et al.* [141] showed that in some cases the mechanism of action of novel antimalarial compounds, identified by high-throughput cell-based screen, can be determined by *in silico* compound activity profiling. Using clustering by historical activities and the guilt-by-association principle, the authors found that the compounds may segregate based on their mechanism of action (e.g., clusters of protein synthesis inhibitors or folic acid antagonists). Using an *in silico* approach, Woynarowski *et al.* [146] found that the targets for AT-specific DNA-reactive antitumor drugs adozelesin and bizelesin, which inhibited the growth of *P. falciparum* at picomolar concentrations, were “super-AT island” regions of the parasite genome. Furthermore, investigation of the essentiality of a reaction in the metabolic network of *P. falciparum* by deleting (knocking-out) such a reaction *in silico* [147], and comparison of reconstructed metabolic network models from the parasite and its human host [148] has revealed essential enzymes from the parasite alone, representing new potential drug targets. As molecular databases of compounds and target structures are becoming larger, more and more computational screening approaches are becoming available, providing new and better insights into *in silico* antimalarial drug discovery [149,150]. Using such computational screening approaches, novel and potent inhibitors of *P. falciparum* plasmepsins [151,152], glutathione-*S*-transferase [153], and DHFR [153] have been identified.

Thus, it is anticipated that with the aid of these new screening technologies, ongoing and new projects, including those at the MMV, will keep the malaria drug discovery and development pipeline full with the next generation of potential malaria chemotherapeutics [46].

### 5.5. Genome-Based (“Targeted”) Drug Discovery

The complete genomes of the predominant malaria parasites *P. falciparum* and *P. vivax*, and the human host are now known. The *P. falciparum* genome sequence comprises of 14 chromosomes containing 5,300 identified genes [154]. Undoubtedly, the completion of these *Plasmodium* spp. genomes, and their comparison with the human genome, provides the opportunity to discover parasite-specific, novel molecular targets for malaria therapy and prevention [155]. The information regarding gene expression and key regulatory components of metabolism in *P. falciparum* is being assembled into various databases, to generate and visualize network models of various cellular pathways and processes [156,157]. Efforts are also underway to characterize the importance of many of these pathways and to predict their potential for pharmacological interventions [158-160]. Furthermore, linking this increase in advancing new genetic information with the expression of drug resistance will greatly assist in identifying sites of drug resistance as well as strategies to improve the treatment [161].

A wealth of information on these themes has been presented in various recent articles [162-168]. The MMV currently has 30 projects in the discovery phase [46]. Out of these, 14 discovery projects are on molecularly defined targets, with defined mechanisms of action, and 16 are on whole-cell activity, and therefore have unknown mechanisms of action. In addition to this, the MMV has a target database that currently contains more than 23 new targets at different stages of screening and validation. The molecular targets in ongoing 14 discovery projects at the MMV include *P. falciparum* cysteine proteases, termed falcipains [169-172], heat shock protein 90 (Hsp90) [173-175], DHFR with new series of inhibitors, a variety of kinases, histone deacetylases (HDAC), and dihydroorotate dehydrogenase (DHODH). Here, we present a summary of the recent key findings related to parasite kinases, HDAC, and DHODH as drug targets. As described below, since these enzymes seem essential for the parasite, they may be highly conserved among *Plasmodium* spp., and mutations in them are likely to be lethal. Therefore, the probability of the parasite becoming resistant to compounds directed against these enzymes should be minimal to none. In addition to these enzyme targets, we summarize key findings related to parasite sequestration receptors/anti-sequestration compounds as another interesting possible drug target.

#### 5.5.1. Kinases

The life cycle of malaria parasites is very tightly regulated, since it passes through a succession of developmental stages that vary in terms of proliferation and differentiation. The successful completion of such a complex life cycle requires a strict control of the cellular machinery that carries out phosphorylation, transcriptional control, post-transcriptional control, and protein degradation. These mechanisms most likely involve fine interactions among various cell-cycle proteins (particularly kinases), which may represent strategic targets for pharmacological interventions [96,104,176].

There are a large number and variety of kinases (“*Plasmodium* kinome”), many of which are clearly distinct from human protein kinases, and thus are considered as targets of choice for antimalarial drug development [176,177]. A serine/threonine protein kinase, casein kinase 2α (PfCK2α), crucial for asexual blood-stage parasites, has been identified as a potential target for antimalarial chemotherapeutic intervention [178]. Among cyclin-dependent protein kinases, Pfmrk is the most attractive target for antimalarial drug development; a variety of inhibitors selectively inhibit Pfmrk at sub-micromolar concentrations [179-181]. Calcium-dependent protein kinase 1 (PfCDPK1), essential for parasite survival, can be inhibited by small-molecule compounds at nanomolar concentrations [182,183]. An orphan protein kinase PfPK7, significant for asexual stage development in humans and oocyst production in mosquitoes, has recently been identified as a lead target [184-187]. In addition, mitogen-activated protein kinase 2 (PfMAP-2), essential for completion of the asexual cycle 188, NIMA-related protein kinase Pfnek-2, predominantly expressed in, and required for transmission of, gametocytes [189], and cAMP-dependent protein kinase (PfPKA), essential for parasite growth and survival [190], hold the promise of being selective and effective targets for the development of new antimalarial drugs.

#### 5.5.2. Histone deacetylases

Histone acetylation plays key roles in regulating gene transcription in both prokaryotes and eukaryotes, the acetylated form inducing gene expression while deacetylation silences genes. A number of HDAC inhibitors are currently in clinical trials, alone or in combination, for the treatment of a variety of cancers, parasitic/infectious diseases, and hemoglobinopathies [191,192]. *Plasmodium falciparum* has at least five putative HDACs, which play a major role in transcriptional regulation of the parasite life cycle [193]. Among these, PfHDAC1 in particular is considered as a promising new antimalarial drug target. A variety of PfHDAC1 inhibitors showed potent *in vitro* antimalarial activity (IC_50_ values in nM range) against CQ-sensitive and CQ-resistant strains of the parasite, and several of the inhibitors were significantly more toxic to the parasites than to mammalian cells [194,195]. Furthermore, an HDAC inhibitor WR301801 (YC-2-88) exhibited cures in *P. berghei*-infected mice at a dose of 52 mg/kg/day orally, when combined with subcurative doses of CQ [196]. Recently, using a modified schizont maturation assay, certain HDAC inhibitors were found to exert potent activity (at nM concentrations) against multidrug-resistant clinical isolates of both *P. falciparum* (n = 24) and *P. vivax* (n = 25) from Papua, Indonesia, suggesting that these inhibitors may be promising candidates for antimalarial therapy in geographical locations where both species are endemic [197].

#### 5.5.3. Dihydroorotate dehydrogenase

Erythrocytic stages of *P. falciparum* seem to maintain an active mitochondrial electron transport chain to serve just one metabolic function: regeneration of ubiquinone required as the electron acceptor for DHODH, an essential enzyme for de novo pyrimidine biosynthesis [198]. For this very reason, PfDHODH is also receiving increasing attention as a new target for antimalarial drug discovery [199,200]. A variety of potent inhibitors of PfDHODH showing strong selectivity for the malarial enzyme over that from the human host have been designed and synthesized, and identified by high-throughput screening [201-204]. Potent activity against *Plasmodium* spp. parasites *in vitro* at nM concentrations, with good correlation between activity against the parasites and the enzyme from the parasites has been observed for a number of these inhibitors [201,205]. Lead optimization of a triazolopyrimidine-based series of inhibitors has identified Genz-667348, which is orally bioavailable, with prolonged plasma exposure, and cures *P. berghei* infection in mice at a total dose of 100 mg/kg/day [201]. Further refinement of the structure-activity relationship within this series is underway, with 2 recent analogs of Genz-667348 undergoing further testing to determine if they represent suitable candidates for preclinical development [201].

#### 5.5.4. Anti-sequestration compounds—An interesting possibility

In addition, new compounds may participate in the combat against falciparum malaria not by directly killing but preventing the parasite from causing severe forms of the disease. One way to achieve this is to inhibit the parasite's ability to sequester. Sequestration occurs because parasite-derived adhesins, expressed on the surface of mature infected erythrocytes, bind to receptors on human cells (often referred to simply as cytoadherence or cytoadhesion). Organ-specific sequestration in brain and placenta play an important role in the pathogenesis of cerebral and placental malaria, respectively [5,206]. *Plasmodium falciparum*-infected erythrocytes have been shown to have the potential for binding to a diverse array of endothelial receptors; however, only two of these receptors, CD36 and intracellular adhesion molecule 1 (ICAM1), have been studied in detail [206]. Chondroitin sulfate A (CSA) has been consistently identified as the dominant placental adhesion receptor [5].

In recent years, some progress has been made in inhibiting cytoadherence that is mediated by these receptors. CD36-mediated cytoadherence can be inhibited *in vitro* by antiretroviral PIs ritonavir and saquinavir [207], and polysaccharides derived from seaweed (carrageenans) [208]. A randomized clinical trial of Thai patients with uncomplicated malaria showed that levamisole, an antihelminth drug, known to block cytoadherence to CD36, when used as an adjunctive therapy with quinine and doxycycline, resulted in increased numbers of early-mid trophozoites in the peripheral blood [209]. It was suggested that levamisole prevented the sequestration of these parasites as they matured from ring stages following treatment [209]. Using the crystal structure of ICAM1, Dormeyer and colleagues [210] found that (+)-epigalloylcatechin gallate, a naturally occurring polyphenol compound in green tea, inhibited cytoadherence to ICAM1 in a dose-dependent manner with two variant ICAM 1-binding parasite lines. Using CSA-expressing CHO-K1 cells and *ex vivo* human placental tissue, Andrews *et al.* [211] showed that two carrageenans and a cellulose sulfate (CS10) were able to inhibit cytoadhesion to CHO-K1 cells as well as human placental tissue; the inhibitory effect on CHO-K1 cells was dose-dependent.

Inhibition of cell adhesion processes is becoming increasingly interesting in the discovery of novel therapeutics [212]. Although our understanding of the molecular mechanisms of parasite adhesion is still incomplete, and many questions remain unanswered [206], the discoveries outlined above open up the possibility of developing therapeutic interventions aimed at blocking or reversing parasite adhesion.

## 6. A Final Note

In these various approaches to: (i) efficient use of existing as well as forthcoming new antimalarial drugs and (ii) discovery of novel antimalarial drugs, one important aspect not discussed here is the pharmacology of antimalarial agents. Since malaria is a blood infection, the response to an antimalarial treatment is likely determined by the concentration of a drug and/or its active metabolite(s) in the blood. Although antimalarial drugs have been in use since the 1930s, until recently, no data were available on the absorption-distribution-metabolism-excretion parameters of these drugs in humans. Over the past 10–15 years, there have been many methodological developments in the area of pharmacology related to antimalarials. Through these advancements, pharmacokinetics and the major metabolic pathways of a number of antimalarial drugs have been elucidated [33,167,213]. However, little emphasis has been placed on understanding the basic mechanisms responsible for the pharmacokinetic and pharmacodynamic behaviors of the major classes of antimalarial drugs in various populations. Particularly, what role does human genetic diversity play in the outcomes of treatment, such as failure, resistance, and side effects, with antimalarial drugs? Since drug combinations are increasingly being considered to successfully treat malaria worldwide, such information is essential to optimize antimalarial drug regimens, particularly new regimens, in order to achieve high treatment effectiveness across all malaria-endemic regions.

## 7. Conclusions

In the battle against malaria, we are currently at a stalemate at best. If and when the resistance to artemisinin drugs further develops and spreads, we will be caught unarmed, without any effective weapon against this deadly parasite. Looking at the antimalarial drug development pipeline, new classes of agents, and, in the meantime, a re-optimization of existing drugs using new creative strategies are needed. Re-optimization of existing drugs through replacement/rotation and combination approaches may prolong their life spans for effective treatment and prophylaxis, until new treatments are found. The future of antimalarial drug discovery lies in innovative thinking and novel areas, some currently under exploration and some yet to be explored. As described in this article, there are a number of different promising approaches to the identification of new antimalarials. Among these approaches, repurposing drugs that are used for other infections or diseases, exploring natural products with medicinal significance (in malaria), and target-based drug discovery are of particular interest. In addition, evolving technologies, such as high-throughput cell-based assays and virtual screening, will certainly be useful in discovering new effective treatments for malaria relatively quickly. We do not have any choice but to explore many or all of these approaches in parallel, until existing drugs with renewed potential, as well as entirely new series of compounds are available for deployment in the field. The discovery of new treatments for malaria, along with their proper execution in the field, will contribute to an important achievement of controlling, and eventually eradicating, this global infectious disease.

## Figures and Tables

**Table 1:** Recent antimalarial drug screening efforts.

**Group**	**Number of compounds screened**	**Number of hits (effective against malaria parasites)**	**Number of new leads (with no reported toxicity to humans)**	**Pre-IND**	**Reference**
Plouffe *et al.*	1,700,000	6,000	530	*	[141]
Gamo *et al.*	2,000,000	13,533	51	*	[142]
Guiguemde *et al.*	309,474	1,300	172	34	[143]
Rottmann *et al.*	12,000	275	17	1	[144]
Rush *et al.*	16,000	17	*	*	[145]
Grimberg *et al.*	33	28	6	3	[88]

* = Ongoing investigations, outcome pending.
